# *Plasmodium* spp. mixed infection leading to severe malaria: a systematic review and meta-analysis

**DOI:** 10.1038/s41598-020-68082-3

**Published:** 2020-07-06

**Authors:** Manas Kotepui, Kwuntida Uthaisar Kotepui, Giovanni De Jesus Milanez, Frederick Ramirez Masangkay

**Affiliations:** 1 Medical Technology, School of Allied Health Sciences, Walailak University, Tha Sala, Nakhon Si Thammarat Thailand; 2 Department of Medical Technology, Institute of Arts and Sciences, Far Eastern University-Manila, Manila, Philippines

**Keywords:** Diseases, Medical research, Signs and symptoms

## Abstract

Mixed *Plasmodium* malaria infections can lead to severe malaria. This systematic review and meta-analysis aimed to explore the prevalence of severe mixed *Plasmodium* malaria infection and to compare it with the prevalence of severe *P. falciparum* malaria mono-infection across the included studies. Original English-language research articles from PubMed, Scopus, and ISI Web of Science were identified and screened. Articles reporting the number of mixed infections and the number of severe mixed infections were used to determine the main outcome of this study, while the number of *P. falciparum* infections and the number of severe *P. falciparum* infections were used to determine the secondary outcome of this study. For the main outcome, the pooled prevalence and 95% confidence interval (CI) of severe mixed infections was analysed using STATA software version 15.0 (Stata Corp, College Station, TX, USA). For the secondary outcome, the rate of severe mixed infections compared to severe *P. falciparum* infections was analysed using the meta-analysis approach, and summary odds ratios (ORs) and 95% CIs were calculated. Random-effects models were used to produce the summary ORs. The Mantel–Haenszel method and calculated I^2^ were also reported to test whether there was heterogeneity among the included studies. Publication bias was also assessed using funnel plots. The meta-analysis of secondary outcomes was conducted using Review Manager 5.3 software (Cochrane Community). A total of 894,561 malaria patients were reported in all 16 included studies. Overall, a pooled analysis showed that 9% (2,006/35,768, 95% CI 7.0–12.0%) of patients with mixed *Plasmodium* infection had severe mixed infection. A meta-analysis of 14 studies demonstrated that patients with mixed *Plasmodium* infection (1,999/35,755) and patients with *P. falciparum* malaria (9,249/294,397) had an equal risk of developing severe malaria (OR 0.93, 95% CI 0.59–1.44). Both mixed infection and *P. falciparum* mono-infection showed a similar trend of complications in which severe anaemia, pulmonary failure, and renal impairment were the three most common complications found. However, patients with mixed infection had a higher proportion of severe anaemia and pulmonary complications than those with *P. falciparum* infection. Moreover, patients with mixed infection had a higher proportion of multiple organ failure than those with *P. falciparum* mono-infection. Mixed *Plasmodium* spp. infections were common but often unrecognized or underestimated, leading to severe complications among these malaria patients**.** Therefore, in routine clinical laboratories, using an accurate combination of diagnostic procedures to identify suspected patients with mixed infections is crucial for therapeutic decisions, prompt treatment, and effective patient management**.**

## Introduction

Human malaria is caused by five species of *Plasmodium* spp. that include *P. falciparum*, *P. vivax*, *P. malariae*, *P. ovale*, and *P. knowlesi*^1^. Molecular methods have demonstrated the existence of two distinct species of *P. ovale*: *P. ovale curtisi* and *P. ovale wallikeri*^2^. *P. knowlesi* naturally occurs in macaques inhabiting forested areas of Southeast Asia and is the fifth species of *Plasmodium* causing human malaria^1,3^. In some areas where more than one species of *Plasmodium* is endemic, mixed *Plasmodium* spp. infections can frequently occur^4^. Mixed *Plasmodium* spp. infections are often unrecognized or underestimated, as a low proportion (2%) is detected by microscopy^5,6^. This might be due to observer error, technical difficulties, and low parasite densities^4^. If the mixed infection is misdiagnosed as a *P. vivax* mono-infection, treatment of *P. vivax* will increase the risk of *P. falciparum* parasitaemia, leading to anti-malarial drug resistance and, eventually, development of severe *P. falciparum* malaria^7^. Therefore, in routine clinical laboratories, the use of the most accurate diagnostic procedures to identify *Plasmodium* species in cases of suspected mixed malaria infection is crucial for therapeutic decisions and management among those patients^7,8^. A research study indicated that mixed *P. falciparum* and *P. vivax* infection led to an increase in the disease severity among children^9,10^. Another study demonstrated that mixed infection with *P. falciparum* and *P. vivax* led to suppression of the severity of *P. falciparum* infection^11^. Although mixed *P. falciparum* and *P. vivax* malaria is common, systematic review and meta-analysis of severe mixed infection has been limited. No recent study has demonstrated the prevalence and differences between mixed *Plasmodium* infection and *P. falciparum* malaria infection. This is very important for physicians to plan therapeutic options and determine the prognostic signs of severity during drug treatment. Therefore, this systematic review and meta-analysis aimed to explore the prevalence of severe *Plasmodium* mixed infection and to compare it with that of severe *P. falciparum* malaria infection across the included studies.

## Methods

### Search strategy

The protocol for this systematic review and meta-analysis followed the Preferred Reporting Items for Systematic Reviews and Meta-Analyses (PRISMA) guidelines (PRISMA Checklist S1). The search strategy started by searching the key terms “(Severe OR complicated OR Complication) AND (Plasmodium OR Malaria) AND (“Mixed infection” OR “Mix infection”)” indexed in PubMed, Scopus, and the ISI Web of Science. The articles published through 25 Jan 2020 were retrieved and reviewed by two independent reviewers. Any discrepancy was judged by the third reviewer (FRM).

### Definition of severe malaria

The major complications of severe mixed malaria were considered to be the same as those defined for *P. falciparum* by the World Health Organization (WHO) and included respiratory distress or acidosis (a base deficit of > 8 meq/L, a plasma bicarbonate of < 15 mM or venous plasma lactate > 5 mM), pulmonary oedema (radiologically confirmed, or oxygen saturation < 92% on room air with a respiratory rate > 30/min), impaired consciousness (a Glasgow Coma Score < 11 in adults or a Blantyre coma score < 3 in children), convulsions (more than two episodes within 24 h), prostration (generalized weakness so that the person is unable to sit, stand or walk without assistance), hypotension/shock (systolic blood pressure < 70 mmHg in children or < 80 mmHg in adults), jaundice [plasma bilirubin > 50 µM/L (3 mg/dL)], severe anaemia (A haemoglobin concentration < 5 g/dL), bleeding/Disseminated Intravascular Coagulation (DIC) (recurrent or prolonged bleeding from the nose, gums, or venepuncture sites; haematemesis or melaena), hyperparasitemia (*P. falciparum* parasitaemia > 10%), and hypoglycaemia [blood or plasma glucose < 2.2 mM (< 40 mg/dL)]^12^. Cerebral malaria, one criterion of severe *P. falciparum* malaria in the former version of the WHO definition, was assigned to the group “impaired consciousness” and described as “impaired consciousness/cerebral malaria” for further analysis and demonstration in the results section.

### Inclusion and exclusion criteria

Original research articles published in the English language were included in the current analysis if they met the following criteria: (1) malaria positivity confirmed by any combination of rapid diagnostic tests (RDTs), microscopy, or polymerase chain reaction (PCR); (2) enrolled both uncomplicated and complicated malaria; (3) the numbers of mixed infections and severe mixed infections were reported, and (4) all complications in the patients with severe mixed infections were reported. Case reports, animal studies, experimental studies, clinical trials, book or book chapters, letters to the editor, editorials, reviews or systematic reviews, conference papers, short surveys, and studies of co-infection of *Plasmodium* with other agents were excluded from the present study.

### Data extraction

For all articles included in the analysis, the following information was extracted: name of the authors, year of publication, country of the participants, duration of the study, the total number of malaria patients, number of severe mixed infections, number of mixed infections, number of severe *P. falciparum* infections, number of *P. falciparum* infections, complications of severe mixed infections, and complications of *P. falciparum* infections. The number of mixed infections and the number of severe mixed infections was used to determine the main outcome of this study, while the number of *P. falciparum* infections and the number of severe *P. falciparum* infections were used to determine the secondary outcome of this study.

### Statistical analysis

For the main outcome, the pooled prevalence and 95% confidence interval (CI) of severe mixed infection was analysed using STATA software version 15.0 (Stata Corp, College Station, TX, USA). For the secondary outcome, the rate of severe mixed infection compared to severe *P. falciparum* infection was analysed using the meta-analysis approach and summary odds ratios (ORs) and 95% CI were calculated. Random-effects models were used to produce summary ORs as described previously^13^. The Mantel–Haenszel method and the calculated I^2^ were also reported to determine whether there was heterogeneity among the included studies. Publication bias was also assessed using funnel plots and Egger's test as described elsewhere^14^. The meta-analysis of the secondary outcomes was conducted using Review Manager 5.3 software (Cochrane Community).

## Results

### Characteristics of the included studies

**Figure 1:**
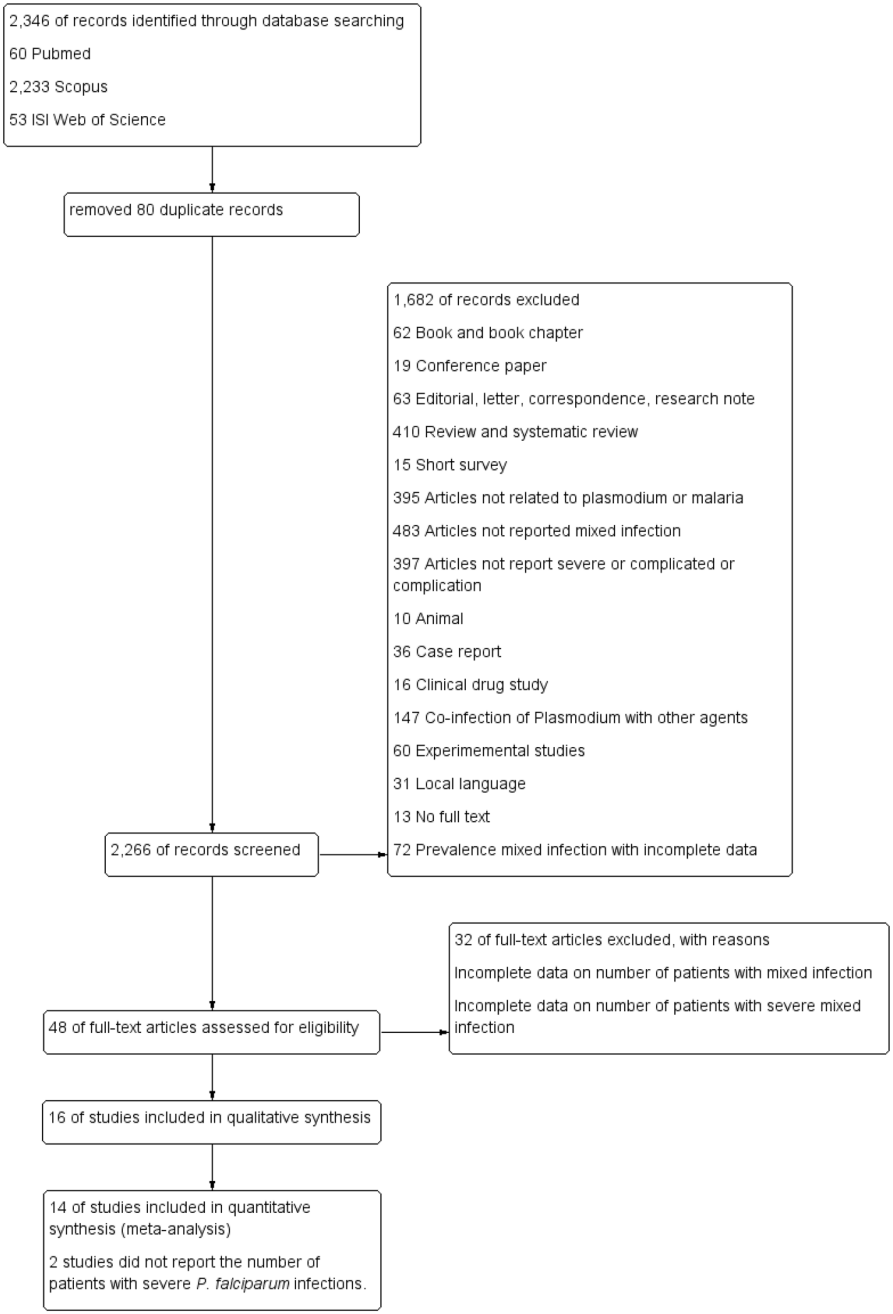
Flow diagram.

The flow diagram of this study according to the PRISMA guidelines is shown in Fig. 1. All 2,346 articles were retrieved from three research databases, including 60 from PubMed, 2,233 from Scopus, and 53 from ISI Web of Science. After 80 duplicated articles were removed, 2,266 articles were processed through title and abstract screening. After title and abstract screening, 48 full-text articles were extensively reviewed, resulting in 16 studies that passed the inclusion and exclusion criteria review. A total of 894,561 malaria patients were reported in all 16 included studies^15–30^. Most of the included studies (56.3%, 9/16) were descriptive studies or cross-sectional observational designs^15–17,20,23,24,26,27,30^. Six studies (37.5%, 6/16) were prospective studies or prospective cohort studies^18,19,21,22,28,29^. One study was a retrospective observational study^25^. The majority of patients in all included studies were infected with *P. vivax* (62.5%, 558,705/894,561), followed by *P. falciparum* (32.9%, 294,397/894,561). Almost all of the included studies reported that *P. falciparum*/*P. vivax* mixed infection was frequently found among those with mixed *Plasmodium* spp. infection. One study reported patients with *P. vivax*/*P. malariae* mixed infection and other types of mixed infection^24^. The majority of malaria patients were identified from the SIVIGILA study conducted in Colombia (547,542 participants)^16^. Half of the studies were conducted in India (9/16, 56.3%), followed by Colombia (3/16, 18.8%). Half of the included studies (8/16, 50%) used combined microscopy techniques and other techniques to confirm the parasite species*.* Four studies used PCR to confirm the *Plasmodium* parasite species (Table 1). The quality of all included studies was shown in Table 2.

**Table 1:** Characteristics of the included studies.

No.	Author	Study area (years of the survey)	Study design	Method for malaria detection	*Plasmodium* sp.	Severe Pf infection (%)	Total malaria	Mixed infection of *Plasmodium* spp.	Number of mixed infection (%)	Severe mixed infection (%)	Complications of mixed infections
1	Chaparro et al.^15^	Colombia Data from SIVIGILA 2010	Descriptive study	Microscopy RDT	*P. falciparum* 32,777 *P. vivax* 82,856 *P.malariae 47*	282 (0.86)	117,108	*Pf/Pv*	1,428 (1.22)	32 (2.24)	Cerebral malaria = 6 Renal impairment = 10 Jaundice = 14 Pulmonary = 1 Unreported = 1
2	Chaparro‑Narváez et al.^16^	Colombia (2007–2013) Data from SIVIGILA	Descriptive study	Microscopy RDT	*P. falciparum* 150,026 *P. vivax* 390,944	1,274 (0.85)	547,542	*Pf/Pv*	6,570 (1.2)	153 (2.32)	Jaundice = 11 Convulsions = 15 Cerebral malaria = 24 Severe anemia = 20 Bleeding/DIC = 10 Shock = 6 Pulmonary = 15
3	Dayanand et al.^17^	India (2013–2016)	Descriptive study	Microscopy	*P. falciparum* 2,456 *P. vivax* 15,334	10 (0.41)	18,936	*Pf/Pv*	1,146 (6.05)	7 (0.61)	Impaired consciousness = 6 Renal impairment = 5 Pulmonary = 6 Hemoglobinuria = 2 Shock = 2 Multi-organ dysfunction = 6
4	Devineni et al.^18^	India (2014–2015)	Prospective study	Microscopy RDT	*P. falciparum* 62 *P. vivax* 114	NA	180	*Pf/Pv*	4 (2.22)	4 (100)	Renal impairment = 4 Pulmonary = 4 Bleeding/DIC = 2 Impaired consciousness = 4 Hyperparasitemia = 4 Hypoglycemia = 2 Death = 4
5	Genton et al.^19^	Papua New Guinea (1997–2004)	Prospective cohort study	Microscopy	*P. falciparum* 6,886 *P. vivax* 1,946 *P.malariae* 328 *P.ovale* 27	261 (3.79)	9,537	*Pf/Pv*	350 (3.67)	24 (6.86)	Pulmonary = 14 Impaired consciousness = 2 Severe anemia = 7
6	Hermansyah et al.^20^	Indonesia (2011–2013)	Descriptive study	Microscopy RDT PCR	*P. falciparum* 8 *P. vivax* 12 *(severe only)*	NA	29	*Pf/Pv*	9 (NA)	3 (33.3)	Cerebral malaria = 2 Convulsion = 1
7	Kochar et al.^21^	India (2007–2008)	Prospective study	Microscopy RDT PCR	*P. falciparum* 555 *P. vivax* 485	274 (44.5)	1,123	*Pf/Pv*	83 (7.4)	44 (53)	Cerebral malaria = 5 Severe anemia = 17 Jaundice = 25 Renal impairment = 6
8	Kochar et al.^22^	India (2007–2008)	Prospective study	Microscopy RDT PCR	*P. falciparum* 185 *P. vivax* 103	79 (42.7)	303	*Pf/Pv*	15 (4.95)	2 (13.3)	Severe anemia = 1 Multiorgan Dysfunction = 1
9	Laman et al.^23^	Papua New Guinea	Descriptive observational study	Microscopy	*P. falciparum* 78 *P. vivax* 3	58 (74.3)	87	*Pf/Pv*	6 (6.9)	4 (66.7)	Cerebral malaria = 1 Convulsion = 1 Severe anemia = 2
10	Langford et al.^24^	Indonesia (2004–2013)	Descriptive study	Microscopy	*P. falciparum* 100,078 *P. vivax* 65,306 *P. ovale* 120 *P. malariae* 5,097	6,361 (6.36)	196,380	*Pf/Pm* 148 *Pv/Pm* 93 (No data on other mixed species)	25,779 (13.1)	1,666 (6.46)	Renal impairment = 84 Pulmonary = 343 Severe anemia = 1,239
11	Limaye et al.^25^	India (2009)	Retrospective observational study	Microscopy RDT	*P. falciparum* 206 *P. vivax* 338	64 (31)	680	*Pf/Pv*	136 (20)	14 (10.3)	Cerebral malaria = 22 Severe anemia = 16 Renal impairment = 14 Pulmonary = 12 Jaundice = 54 Shock = 1 Death = 14
12	Medina-Morales et al.^26^	Colombia (2013)	Descriptive cross-sectional study	Microscopy	*P. falciparum* 17 *P. vivax* 313	3 (17.6)	349	*Pf/Pv*	19 (5.4)	3 (15.8)	Pulmonary = 2 Severe anemia = 1
13	Mittal et al.^27^	India (2011)	Descriptive study	Microscopy RDT	*P. falciparum* 66 *P. vivax* 128	52 (78.8)	198	*Pf/Pv*	4 (2)	4 (100)	Cerebral malaria = 1 Severe anemia = 1 More than 1 complications = 2
14	Mohapatra et al.^28^	India (2007–2009)	Prospective study	Microscopy RDT	*P. falciparum* 770	440 (57.1)	888	*Pf/Pv*	118 (13.3)	21 (17.8)	Cerebral malaria = 4 Jaundice = 2 Severe anemia = 8 More than 1 complications = 7
15	Nayak et al.^29^	India (2010–2011)	Prospective study	Microscopy RDT PCR	*P. falciparum* 147 *P. vivax* 459	68 (46.3)	642	*Pf/Pv*	36 (5.6)	12 (33.3)	Severe anemia = 3 Pulmonary = 6 Cerebral malaria = 1 Hypoglycemia = 1 Renal impairment = 1 Bleeding = 5 More than 1 complications = 3
16	Punnath et al.^30^	India (2013–2015)	Descriptive cross-sectional study	Microscopy	*P. falciparum* 150 *P. vivax* 364	23 (15.3)	579	*Pf/Pv*	65 (11.2)	13 (20)	Shock = 3 Pulmonary = 2 Renal impairment = 1 Jaundice = 4 Severe anemia = 5 Cerebral malaria = 1 More than 1 complications = 9
	Total	India = 9/16 (56.3%) Colombia = 3/16 (18.8%) Papua New Guinea = 2/16 (12.5%) Indonesia = 2/16 (12.5%)	Descriptive study = 9/16 (56.3%) Prospective study = 6/16 (37.5%) Retrospective observational study = 1/16 (6.3%)	Microscopy alone = 6/16 (37.5%) Microscopy with other technique = 8/16 (50%)	*P. falciparum* 294,397 (32.9%) *P. vivax* 558,705 (62.5%) *P. malariae* 5,472 (0.6%) *P. ovale 147* (0.02%)	9,222 (3.13)	894,561		35,768 (4)	2,006 (6.7)	Cerebral malaria/impaired consciousness = 79 (3.94%) Renal impairment = 125 (6.23%) Jaundice = 110 (5.48%) Pulmonary = 420 (20.9%) Convulsions = 17 (0.85%) Severe anemia = 1,320 (65.8%) Bleeding/DIC = 17 (0.85%) Shock = 12 (0.6%) Hyperparasitemia = 4 (0.2%) Hypoglycemia = 3 (0.15%) Death = 18 (0.9%) More than 1 complications = 27 (13.1%)

**Table 2:** Quality of the included studies.

No.	References	Selection				Compatibility	Exposure		
		Is the case definition adequate?	Representativeness of the cases	Selection of controls	Definition of controls		Ascertainment of exposure	Same method of ascertainment for cases and controls	Non-response rate
1	Chaparro et al.^15^	*	*	*	*	**	*	*	*
2	Chaparro‑Narváez et al.^16^	*	*	*	*	**	*	*	*
3	Dayanand et al.^17^	*	*	*	*	**	*	*	*
4	Devineni et al.^18^	*	*			**	*	*	*
5	Genton et al.^19^	*	*	*	*	**	*	*	*
6	Hermansyah et al.^20^	*	*			**	*	*	*
7	Kochar et al.^21^	*	*	*	*	**	*	*	*
8	Kochar et al.^22^	*	*	*	*	**	*	*	*
9	Laman et al.^23^	*	*	*	*	**	*	*	*
10	Langford et al.^24^	*	*	*	*	**	*	*	*
11	Limaye et al.^25^	*	*	*	*	**	*	*	*
12	Medina-Morales et al.^26^	*	*	*	*	**	*	*	*
13	Mittal et al.^27^	*	*	*	*	**	*	*	*
14	Mohapatra et al.^28^	*	*	*	*	**	*	*	*
15	Nayak et al.^29^	*	*	*	*	**	*	*	*
16	Punnath et al.^30^	*	*	*	*	**	*	*	*

### The prevalence of mixed *Plasmodium* spp. infection

**Figure 2:**
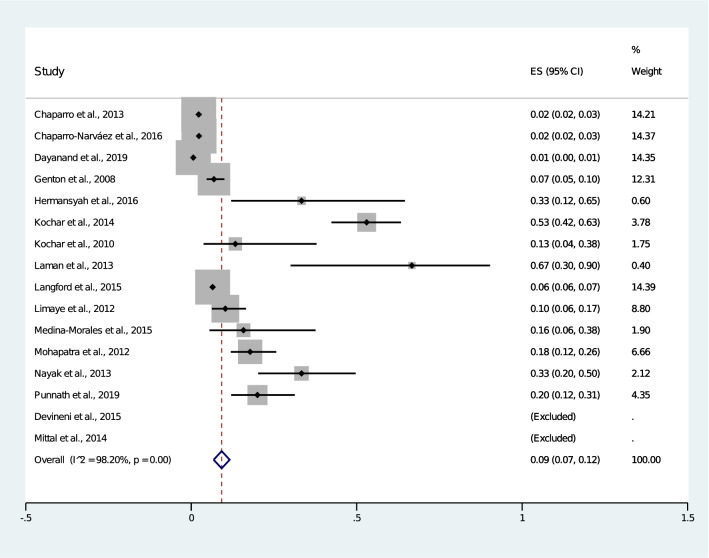
Pooled prevalence of severe mixed infection.

**Figure 3:**
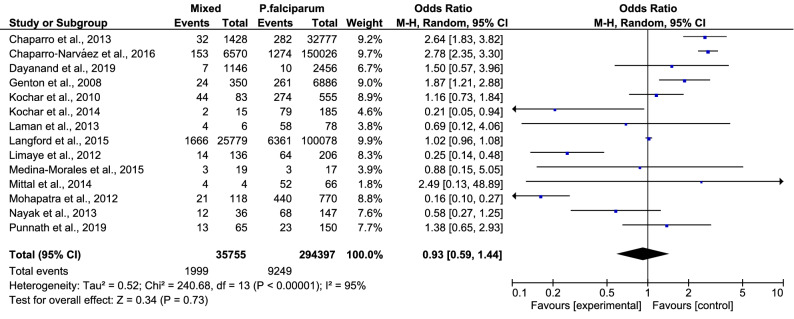
Mixed infection versus *P. falciparum* infection.

Overall, the pooled analysis showed that 9% (2,006/35,768, 95% CI 7.0–12.0%) of patients with mixed *Plasmodium* infection had a severe mixed infection (Fig. 2). There was statistical heterogeneity (I^2^: 98.2%) among the included studies, suggesting a high level of heterogeneity between studies, so random-effects models were used to produce the summary ORs in the present meta-analysis. Among the 16 included studies, only 14 studies were used to perform the meta-analysis, as two studies by Devineni et al., 2015 and Hermansyah et al., 2016 did not report the number of patients who had severe *P. falciparum* infections. The meta-analysis of these 14 studies demonstrated that patients with mixed *Plasmodium* infection (1,999/35,755) and those with *P. falciparum* mono-infection (9,249/294,397) had an equal risk of developing severe malaria (OR 0.93, 95% CI 0.59–1.44) (Fig. 3)^15–17,19,21–30^. Three studies demonstrated that patients with mixed infection had a significantly lower risk of developing severe malaria than patients with *P. falciparum* mono-infection^15,16,19^. Three studies demonstrated that patients with a mixed infection had a significantly higher risk of developing severe malaria than patients with a *P. falciparum* mono-infection^21,25,28^.

**Figure 4:**
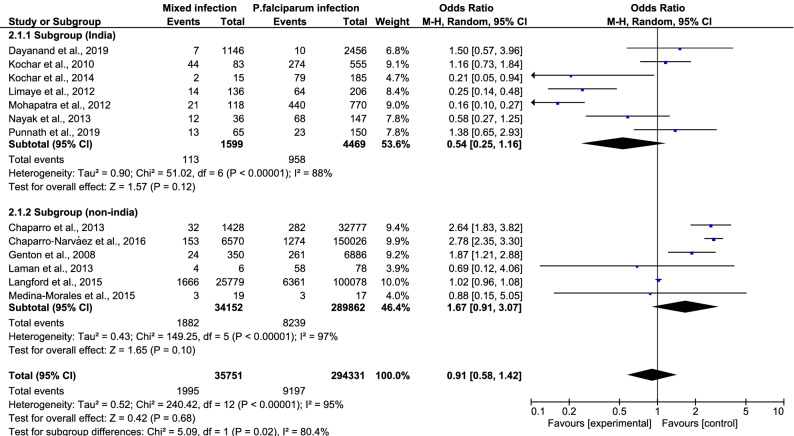
Subgroup analysis of India.

**Figure 5:**
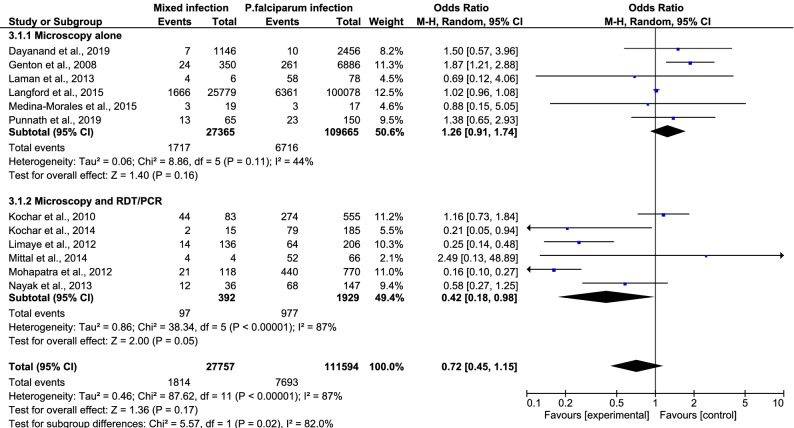
Subgroup analysis of diagnostic technique.

In a subgroup analysis comparing the results from India and non-India areas in 13 studies, the pooled analysis showed that patients with mixed *Plasmodium* spp. infection and patients with *P. falciparum* mono-infection had an equal risk of developing severe malaria (OR 0.91, 95% CI 0.58–1.42) (Fig. 4). There was a subgroup difference (*P *value = 0.02, I^2^ = 80.4%) in this subgroup analysis, indicating that the study area (India and non-India) was one source of heterogeneity in the present study. Further stratification by diagnostic technique (microscopy alone and microscopy with other techniques) also showed that patients with mixed infection had an equal risk of developing severe malaria compared to those with *P. falciparum* mono-infection (OR 0.72, 95% CI 0.45–1.15) (Fig. 5). Once again, there was a subgroup difference (*P *value = 0.02, I^2^ = 82%) in this subgroup analysis, indicating that diagnostic technique (microscopy alone and microscopy with other techniques) was also a source of heterogeneity in the present study.

### Complications of severe mixed infection

**Figure 6:**
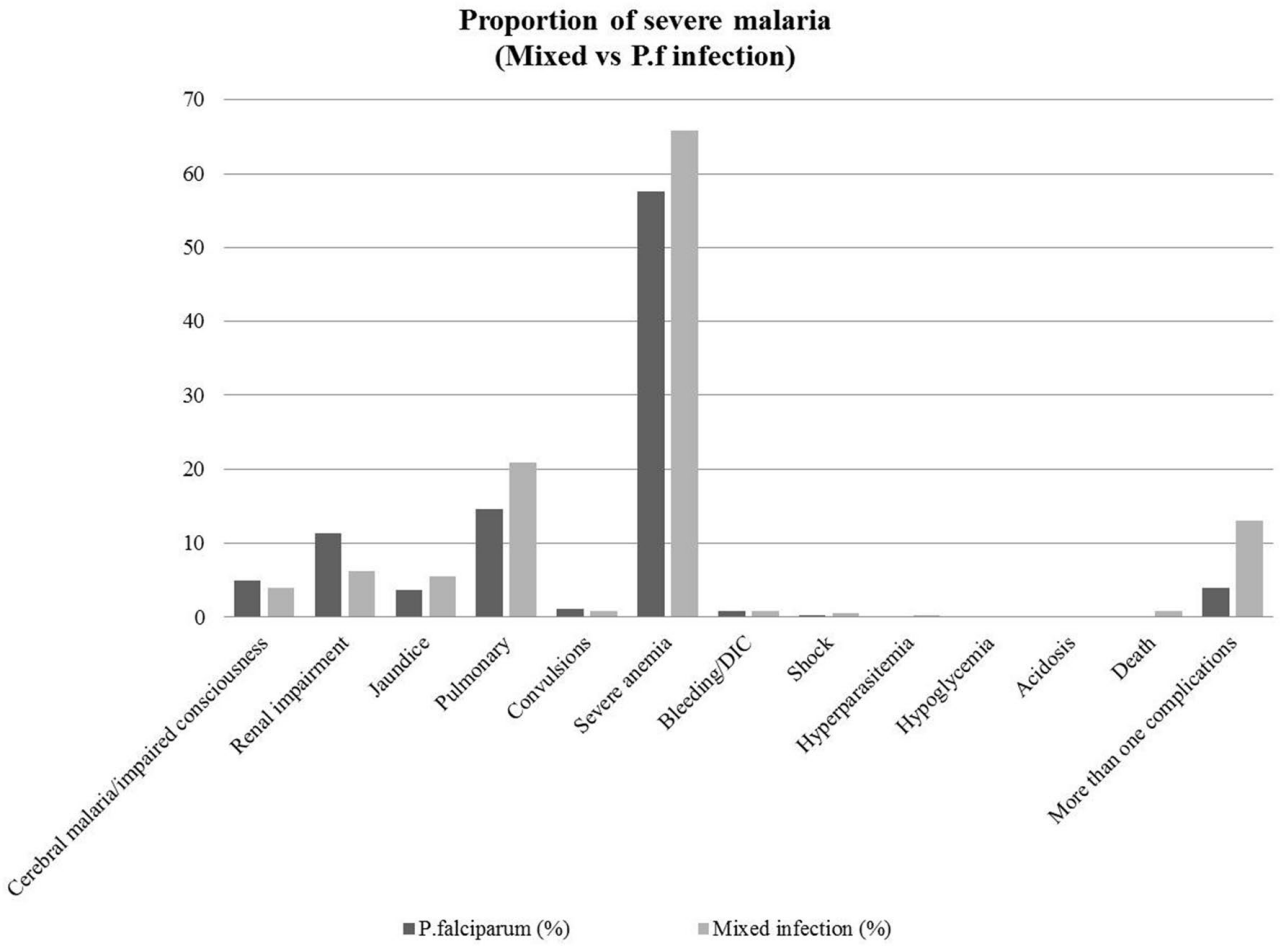
The proportion of severe mixed malaria infection and severe *P. falciparum* mono-infection.

**Figure 7:**
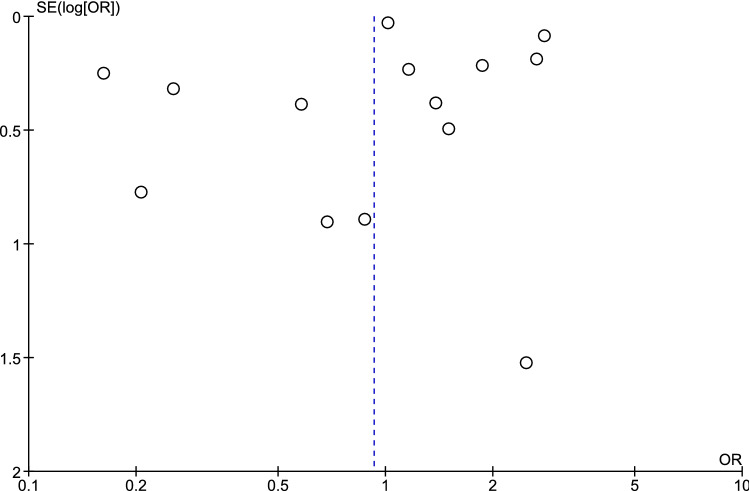
Funnel plot.

Common severe complications found in patients with mixed malaria infection were severe anaemia (65.8%, 1,320/2006), pulmonary failure (20.9%, 420), renal impairment (6.23%, 125), jaundice (5.48%, 110), cerebral malaria/impaired consciousness (3.94%, 79), convulsions (0.85%, 17), bleeding/DIC (0.85%, 17), shock (0.6%, 12), hyperparasitemia (0.2%, 4), hypoglycaemia (0.15%, 3), and more than one complication (13.1%, 27/2006). The mortality rate of severe mixed infection was 0.9% (18/2006). The most common severe complications of *P. falciparum* mono-infection were severe anaemia (57.6%, 5,312/9,222), pulmonary complications (14.6), and renal impairment (11.4%). For all complications, the proportions of severe mixed malaria infection and severe *P. falciparum* infection are shown in Fig. 6. Both mixed infection and *P. falciparum* mono-infection showed similar trends of severe complications by severe anaemia, pulmonary failure, and renal impairment, which were the three most common complications found in this study. Patients with mixed infection had a higher proportion of severe anaemia (65.8% vs 57.6%) and pulmonary complications (20.9% vs 14.6%) than those with *P. falciparum* mono-infection. Patients with mixed infection (13.1%) had a higher proportion of multiple organ failure than those with *P. falciparum* mono-infection (3.95%). The publication bias among studies was assessed by funnel plots (Fig. 7) and Egger's test for small-study effects. The result of Egger's test indicated that no publication bias was found in the present study (*P* value = 0.857, t = 0.18, 95% CI = − 2.57–3.04).

## Discussion

The present study showed a high prevalence of severe mixed *Plasmodium* infection across the included studies (9%), demonstrating for the first time, to our knowledge, that mixed infection can cause a high rate of severe malaria. Although the mixed malaria prevalence was predominantly due to *P. falciparum*/*P. vivax* infection, the prevalence of non-*P. falciparum*/*P. vivax* mixed infections*,* such as *P. falciparum*/*P. malariae* mixed infections was also reported in our study, but they were not shown in their literature^24^. This high prevalence of severe mixed malaria partly explains why malaria remains one of the leading causes of morbidity and mortality worldwide despite available interventions, public health control, and management employed. These findings suggested that there is a need for continued detection and monitoring of mixed infection using species-specific RDTs in combination with routine microscopy, or even using PCR as soon as possible, to move towards malaria elimination and to protect against severe malaria resulting in death. A previous study indicated that the severity of mixed *P. falciparum*/*P. vivax* infection occurred when *P. vivax* superinfection occurred over an existing *P. falciparum* infection. However, *P. falciparum* superinfection over an existing *P. vivax* infection results in a lower risk of severe malaria^28^. The present study demonstrated the equal prevalence of severe mixed malaria infection compared to severe *P. falciparum* mono-infections across the included studies. However, when considering individual studies, it was demonstrated that three of our included studies reported that patients with mixed malaria infection had a significantly lower risk of developing severe malaria than patients with *P. falciparum* mono-infection^15,16,19^. These results were consistent with results from a previous study conducted in Thailand, which observed that severe malaria was less common among patients with mixed infections compared to those with *P. falciparum* mono-infection^31^. However, three other included studies demonstrated that patients with mixed infections had a significantly higher risk of developing severe malaria than patients with *P. falciparum* mono-infections^21,25,28^. This could be because mixed infections are often unrecognized or underestimated by microscopists^32,33^, leading to treatment failure, anti-malarial drug resistance, and the development of severe *P. falciparum* malaria^7^. Previous studies also demonstrated that age was associated with mixed infection^34,35^. They found that children under two years of age had a lower frequency of mixed *Plasmodium* malaria compared to those at an older age. They suggested that maternal antibodies could be the source of mixed infection malaria protection^34^.

The major complications of severe malaria defined by the World Health Organization (WHO) included respiratory distress, acidosis, pulmonary oedema, death, impaired consciousness, convulsions, prostration, hypotension/shock, jaundice, severe anaemia, bleeding/DIC, hyperparasitemia, and hypoglycaemia^12^. The present study demonstrated that 9% of severe malaria was caused by mixed infection, whereas a previous study showed that severe malaria accounts for approximately 5% of total malaria-infected patients^36^. The mortality rate of severe mixed malaria in the present study was 0.9%, which was consistent with the case fatality rate in previously reported *P. falciparum* malaria mono-infection (0.6–3.8%)^4^. The present study also indicated that both mixed infection and *P. falciparum* mono-infection showed similar trends of complications in which severe anaemia, pulmonary failure, and renal impairment were the three most common complications. However, patients with mixed infection had a higher proportion of severe anaemia and pulmonary complications than those with *P. falciparum* mono-infection. Moreover, patients with mixed infection had a higher proportion of multiple organ failure than those with *P. falciparum* mono-infection. A study in Thailand indicated that mixed *P. falciparum*/*P. vivax* infection could reduce the risk of severe anaemia among patients with falciparum malaria by cross-species immunity^37^. In Southeast Asia, other possible reasons behind the reduction of the risk for severe anaemia among patients with malaria infections were haemoglobinopathies and enzymatic deficiencies^38^. Haemoglobinopathies related to the reduced risk of malaria infections or reducing the risk of severe malaria included sickle cell traits^39^, haemoglobin C^40^, haemoglobin E^41^, and thalassemia^40^. Enzymatic deficiencies related to the reduced risk of malaria infections include glucose-6-phosphate dehydrogenase (G6PD) deficiency^42^ and pyruvate kinase deficiency^43^. In addition, individuals with blood type O were less susceptible to severe malaria than individuals who were not blood type O^44^. The expression of the host RBC surface protein called Duffy antigen receptor for chemokines (DARC) has been shown to protect against malaria infections^38^. Moreover, altered RBC morphologies such as Southeast Asian ovalocytosis (SAO) could reduce the risk of malaria infection or severe malaria^45,46^.

The included studies conducted in Papua New Guinea (1997–2004) demonstrated that mixed infection caused more severe anaemia than did the *Plasmodium* mono-infection alone^19^. The results of our study were also consistent with the results of studies in India^9^ and Indonesia^47^ that reported a high prevalence of severe anaemia among patients with mixed infections. The higher proportion of severe mixed infection than that of *P. falciparum* and *P. vivax* mono-infection was due to mixed infection having higher parasite densities^19^.

The present study had limitations. First, there was a high level of heterogeneity across the included studies. Second, except for the area of the study (India and non-India) and diagnostic method, the source(s) of heterogeneity could not be explored due to the incomplete data among the included studies. Third, a limited number of studies met the criteria for inclusion because many publications included patients with severe complications and infections with etiologic agents other than malaria. Fourth, most of the included studies used microscopy for malaria detection, which might have led to missed detection of *Plasmodium* mixed infections. The analysis of mixed-species infections compared with *P. falciparum* mono-infections needs to be carefully interpreted as it is highly likely to be confounded by a proportion of undiagnosed mixed infections in the *P. falciparum* mono-infection groups. Fifth, a large number of additional factors related to transmission intensity, host immunity, and vectors that likely influenced the large variance seen in the mixed-*Plasmodium* species infections could not be taken into account because of the inherent data limitations from each study. Lastly, the present Review submits analysis of data which is relevant for the asexual blood stages of *Plasmodium* spp. infections resulting to severe manifestation and does not take into account hypnozoites and/or submicroscopic co-infections.

## Conclusion

Mixed *Plasmodium* spp. infections are common but often unrecognized or underestimated, leading to severe complications among malaria patients. Therefore, in routine clinical laboratories, using an accurate combination of diagnostic procedures or repeat blood film examinations by microscopists to identify mixed infection in suspected patients is crucial for therapeutic decisions, prompt treatment, and effective management among those patients.

## Supplementary information

Supplementary information

## Data Availability

The datasets used during the current study are available from the corresponding author based on reasonable request.

## Abbreviations

RDT: Rapid diagnostic test
PCR: Polymerase chain reaction
CI: Confidence interval
ORs: Odds ratios
